# Case report: Primary pleural squamous cell carcinoma in a 68-year-old male

**DOI:** 10.3389/fsurg.2022.953989

**Published:** 2022-09-02

**Authors:** Chuan Zhong, Haining Zhou, Ramón Rami-Porta, Yunfei Zhao

**Affiliations:** ^1^ Department of Thoracic Surgery, Suining Central Hospital, Suining, China; ^2^ Department of Thoracic Surgery, Hospital Universitari Mútua Terrassa, University of Barcelona, Terrassa, Spain; ^3^ Department of Pathology, Suining Central Hospital, Suining, China

**Keywords:** primary pleural squamous cell carcinoma, surgical resection, radiotherapy, pulmonary bullae, a promising therapeutic approach

## Abstract

**Introduction:**

Primary pleural squamous cell carcinoma (PPSCC) is a sporadic disease that is rarely reported in the literature. Due to its low incidence, the pathogenesis of PPSCC is unknown.

**Case summary:**

We report a case of a 68-year-old male with PPSCC and sizable pulmonary bullae. Two months after complete resection of both lesions, a total dose of 50 Gy radiotherapy was administered over the operative field. After more than a year of follow-up, the patient is in steady condition without any sign of recurrence.

**Conclusion:**

Since PPSCC is rarely reported, our case proposed that complete surgical resection combined with radiotherapy may be a promising therapeutic approach.

## Introduction

Among epithelial tumors derived from the pleura, the most common are mesothelial tumors such as malignant mesothelioma (1). However, there is a rarely reported epithelial tumor that originates from the pleura—primary pleural squamous cell carcinoma (PPSCC) (2). Very few case reports have reported the incidence of this disease, and its pathogenic mechanism is poorly understood (3, 4). Therefore, there is no standard treatment for PPSCC. Herein, we report on a patient with a PPSCC case accompanied by pulmonary bullae, who completely recovered after receiving complete resection and radiotherapy. Our report will provide a reference for the treatment of PPSCC combined with pulmonary bullae.

## Case presentation

**Figure 1:**
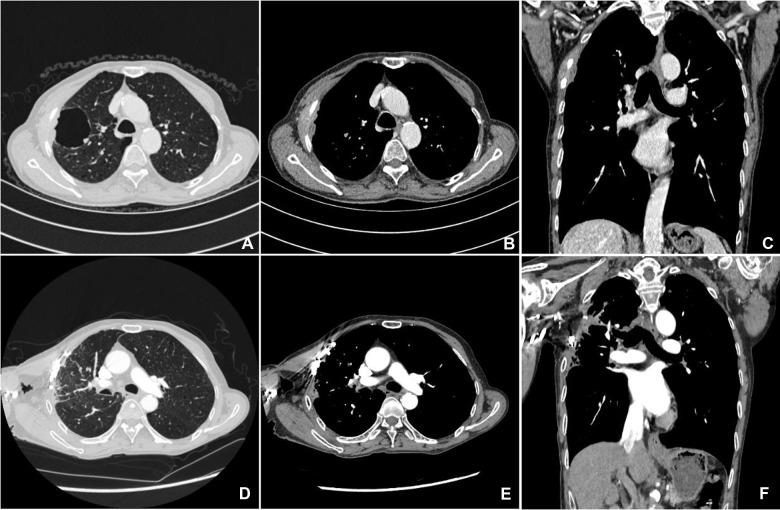
Preoperative chest CT scan (**A–C**); postoperative chest CT scan after more than a year of follow-up (**D–F**).

**Figure 2:**
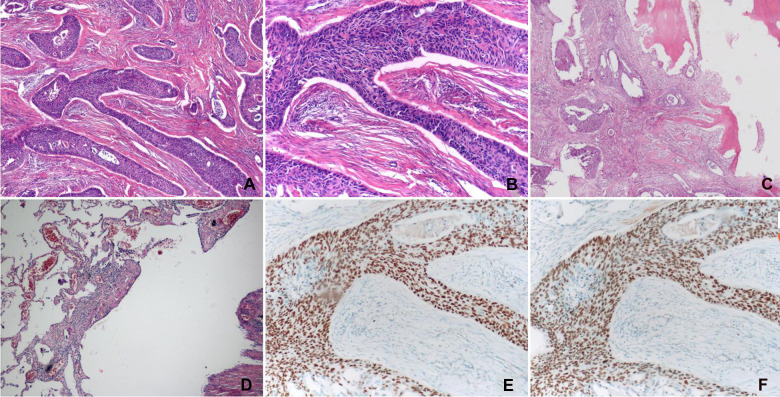
(**A**) The neoplastic cells were arranged in the form of nests with different sizes and showed various intercellular bridges (H&E stain, magnification ×40). (**B**) The cells were round or ovoid, with abundant cytoplasm, large nuclei, apparent nucleoli, and intracellular keratinization in focal areas (H&E stain, magnification ×100). (**C**) Squamous cell carcinoma invading the adjacent ribs (H&E stain, magnification ×40). (**D**) Pulmonary bullae with chronic inflammation (H&E stain, magnification ×40). (**E,F**) Immunohistochemical staining of neoplastic cells was performed with the use of antisera against p40 and p63, respectively, which showed evident nuclear positivity for both (**E**) p40 and (**F**) p63.

A 68-year-old male complained of right-sided chest pain for 4 months with no other symptoms. He did not have history of echinococcosis, tumor, or tuberculosis. However, he had been smoking 20 cigaretes and drinking 100 g of alcohol daily for 40 years. Physical examination revealed no relevant findings. Routine blood and urine tests showed no anemia or proteinuria, respectively. Computed tomography (CT) revealed thickened pleura on the right anterolateral aspect of the chest wall, a soft tissue mass adjacent to the chest wall surrounding part of the 3rd and 4th ribs, a big pulmonary bulla with a size of 4.4 cm × 5.1 cm, and normal mediastinal lymph nodes (Figures 1A–C). We recommended PET/CT to the patient, who declined due to financial reasons. No positive lesion was found after a series of examinations were performed, including cranial magnetic resonance imaging (MRI), contrast-enhanced neck and abdominal CT scans, bronchoscopy, and whole body radionuclide bone scintigraphy. Because all these examinations did not reveal any evidence of regional or distant spread, invasive staging of the mediastinum by mediastinoscopy or of the pleural space by video-thoracoscopy was not indicated. The initial diagnosis was localized mesothelioma with pulmonary bulla. The lesion was deemed completely resectable, and the patient underwent right thoracotomy through the third intercostal space. The intraoperative exploration revealed that the mass of the subcutaneous tissue had a size of 4 cm × 5 cm, accompanied by a pleural lesion with a size of 5 cm × 6 cm. The internal margin of the lesion was attached to the edge of a small part of the adjacent bulla, but the wall of most of the remaining bulla was intact. Intraoperative frozen biopsy of the mass reported a malignant tumor, compatible with squamous cell carcinoma. Therefore, the tumor and bullae were entirely removed. The resection included the chest wall, part of the third and fourth ribs, tissues of the intercostal space and pleura, and a wedge resection of the pulmonary bulla. Appropriate nickel–titanium memory alloy embracing device was fixed on the third and fourth rib stumps to avoid the local collapse of the chest wall. The postoperative histopathological examination confirmed that the tumor was a squamous cell carcinoma that had invaded the adjacent ribs. The wall of the pulmonary bulla showed chronic inflammation (Figures 2A–D). The immunohistochemistry staining showed positive results of P63 and P40 and negative results of TTF1, napsin A, WT1, D2-40, c-kit, and calretinin (Figures 2E,F). Because there were no signs of tumor in other organs, such as lung, mediastinum, head, neck, and abdomen, it was diagnosed as a primary pleural tumor (5). There were no postoperative complications. Two months after the surgery, the patient returned and received radiotherapy over the operative field, with a total dose of 50 Gy. After more than a year of follow-up, no sign of tumor recurrence was detected (Figures 1D–F). The patient was satisfied to our treatment.

## Discussion

PPSCC is an extremely rare disease, with sporadic cases reported in the literature. Due to its low incidence, the pathogenesis of PPSCC remains largely unknown (4). Emerging evidence indicated that inflammation played a critical role in tumorigenesis (6). When tissues have chronic inflammation, the susceptibility to cancer will be promoted. In contrast, the long-term application of non-steroidal anti-inflammatory drugs will decrease the risk of several cancers (7). Previous studies suggested that chronic inflammation may be a pathogenic factor for PPSCC, which was supported by the fact that some patients with PPSCC had empyema, pleurocutaneous fistula (8), extrapleural pneumothorax without fistula in the treatment of tuberculosis (8), tuberculosis (9), or bronchopleural fistula caused by bronchiectasis (10). In this case, the presence of a pulmonary bulla with chronic inflammation in the vicinity of the tumor might promote neoplastic changes of the adjacent pleura, resulting in the development of squamous cell carcinoma. However, we are aware that this finding may be simply coincidental in our patient. Meanwhile, genetic deficiency may also account for the occurrence of PPSCC. Yoshida et al. reported that a 33-year-old female with extensive PPSCC had a deficiency of *SMARCB1/INI1* gene, which was a tumor suppressor gene. The loss of *SMARCB1* was associated with malignant tumors of the kidney, gastrointestinal tract, pancreas, and uterus (5). As for the diagnosis of PPSCC, there are no symptom which is specific for PPSCC. CT or PET–CT are typical approaches for the diagnosis of PPSCC. The preoperative biopsy using transthoracic Tru-Cut needle biopsy, is an approach for the diagnosis of pleural tumors. However, the diagnostic accuracy rate is not satisfactory (3). In this case, the differential diagnosis included focal mesothelioma, primary or secondary chest wall tumors, pleural metastasis, and thymic carcinoma, among which mesothelioma had the highest incidence rate. However, this potential was excluded by the diffuse expression of p40 and p63 and the negative expression of WT1, calretinin, and D2-40 in the tumor (4, 5, 11). Besides, primary chest wall tumors arise from muscle, fat, blood vessel, nerve sheath, cartilage, or bone of the chest wall. Therefore, the postoperative histopathological report of squamous cell carcinoma excluded the possibility of primary chest wall tumors. Furthermore, there was no tumor history and radiological evidence indicating that this tumor originated from lung, mediastinum, head, neck, or abdomen. Therefore, secondary chest wall tumors and pleural metastasis were further excluded. Although most thymic carcinomas were found to be squamous cell carcinoma, the tumor in the present case did not locate in the mediastinum and lacked the expression of CD117 (c-kit) that was a highly specific biomarker for thymic cancer (5). Consequently, we made the diagnosis of PPSCC.

Resection is a recommended approach for local tumors (4). Rüttner et al. reported that two PPSCC patients received surgery and were free of disease for 3 and 5 years after the operation, respectively (8). On the other hand, Prabhakar et al. reported that one PPSCC patient died from massive hemorrhage 5 months after resection. However, local or distant tumor recurrence could not be ruled out since necropsy was not performed (10). Sigala et al. reported that a patient with extensive PPSCC was treated with nivolumab after six cycles of cisplatinum and docetaxel combined with regional radiotherapy. Even though some tumor responses were observed, the tumor progressed locally and distantly, and the patient died 2 years after diagnosis (12). Besides, Yoshida et al. reported that a patient with extensive PPSCC died 10 months after diagnosis even treated with intensive chemotherapy (5). Therefore, chemotherapy might not be efficacious for PPSCC. In this case, the patient received complete resection and radiotherapy, and the follow-up indicated no tumor recurrence. Although no evidence suggested that radiotherapy could improve the prognosis of patients with PPSCC, our case report suggest that the combination of resection and adjuvant radiotherapy may be useful in the management of these patients.

## Conclusion

We reported a rare case of PPSCC combined with pulmonary bulla in an elderly patient. Complete surgical resection combined with radiotherapy achieved more than 1 year of disease-free survival, which may provide a reference for the treatment of PPSCC.

## Data Availability

The original contributions presented in the study are included in the article/Supplementary Material, further inquiries can be directed to the corresponding authors.

## References

[1] Galateau-Salle F Churg A Roggli V Travis W.D, The World Health Organization committee for Tumors of the Pleura. The 2015 World Health Organization classification of tumors of the pleura: advances since the 2004 classification. J Thorac Oncol. (2016) 11(2):142–54. 10.1016/j.jtho.2015.11.005 26811225

[2] Franke M Chung HD Johnson FE. Squamous cell carcinoma arising from the pleura after pneumonectomy for squamous cell carcinoma of the lung. Am J Surg. (2010) 199:e34–5. 10.1016/j.amjsurg.2009.05.004 20359562

[3] Lin XM Chi C Chen J Liu Y Li P Yang Y. Primary pleural squamous cell carcinoma misdiagnosed as localized mesothelioma: a case report and review of the literature. J Cardiothorac Surg. (2013) 8:50. 10.1186/1749-8090-8-50 23497477 PMC3639154

[4] Ronchi A Cozzolino I Montella M Vicidomini G Morgillo F Della Corte C M Primary pleural squamous cell carcinoma: a diagnostic challenge. Cytopathology. (2018) 29:205–7. 10.1111/cyt.12498 29159961

[5] Yoshida K Fujiwara Y Goto Y Kohno T Yoshida A Tsuta K The first case of SMARCB1 (INI1)-deficient squamous cell carcinoma of the pleura: a case report. BMC Cancer. (2018) 18:398. 10.1186/s12885-018-4321-x 29625594 PMC5889546

[6] Balkwill F Coussens LM. Cancer: an inflammatory link. Nature. (2004) 431(7007):405–6. 10.1038/431405a 15385993

[7] Gupta RA Dubois RN. Colorectal cancer prevention and treatment by inhibition of cyclooxygenase-2. Nat Rev Cancer. (2001) 1(1):11–21. 10.1038/35094017 11900248

[8] Rüttner JR Heinzl S. Squamous-cell carcinoma of the pleura. Thorax. (1977) 32:497–500. 10.1136/thx.32.4.497 929492 PMC470658

[9] Garty I Straussberg R Flatau E. Extraosseous 99mTc-MDP uptake in squamous cell carcinoma of the pleura. Eur J Nucl Med. (1987) 12:589–91. 10.1007/BF00284531 3582396

[10] Prabhakar G Mitchell IM Guha T Norton R. Squamous cell carcinoma of the pleura following bronchopleural fistula. Thorax. (1989) 44:1053–4. 10.1136/thx.44.12.1053 2617446 PMC1020887

[11] Ordóñez NG. Application of immunohistochemistry in the diagnosis of epithelioid mesothelioma: a review and update. Hum Pathol. (2013) 44(1):1–19. 10.1016/j.humpath.2012.05.014 22963903

[12] Sigala I Alevizopoulos N Elefteriou K Gianniou N Kalomenidis I. Primary squamous cell carcinoma of the pleura treated with nivolumab. Respirol Case Rep. (2020) 8:e00516. 10.1002/rcr2.516 32042427 PMC7001115

